# Severe pica in long-term schizophrenia, a case report

**DOI:** 10.1192/j.eurpsy.2021.1856

**Published:** 2021-08-13

**Authors:** M. Martínez Querol, M. Lado Codesido

**Affiliations:** Psychiatry, Hospital Universitario Donostia, Donostia-San Sebastian, Spain

**Keywords:** schizophrénia, Pica, eating disorder

## Abstract

**Introduction:**

Pica is a strange eating disorder that consists of eating non-nutritive substances, inappropriate to the developmental level/ cultural normative of the individual. The prevalence is not widely studied, but might occur in the context of other mental disorders, such as schizophrenia, that hinders the management and treatment of these patients.

**Objectives:**

To report a severe pica in a patient with late schizophrenia, and highlight the impact this syndrome might cause on the life of these patients.

**Methods:**

We present a case of a 65 year-old-woman with schizophrenia attended in the emergency area for dysphagia due to the intake of a metal washer. Reviewing the patient medical history, an early and severe schizophrenia was described. Within years, disorganization and residual symptoms have become the main disabilities, developing a pica eating disorder with preference in greater metal objects (images are included).

**Results:**

The management has been hindered due to the consequences of pica eating disorder. The patient describes an unstoppable urge to make the intake when she sights wide metallic objects (batteries, docks, washers…). Different antipsychotic drugs have been used, combined with psychotherapy and family education. Despite this, the patient has required multiple attentions in the emergency area due to esophagueal impaction, intestinal obstruction, perforation and peritonitis, that have led to countless surgeries and hospitalizations.

**Conclusions:**

Pica can become a highly dysfunctional syndrome that may lead to severe organic and life impact. The comorbidity with schizophrenia is understudied, but further investigation might be useful to show up specific management strategies of these patients.

**Disclosure:**

No significant relationships.

